# An Optimized Image-Processing Algorithm for Semi-Automated Measurement of Attached Cavities in High-Speed Flow Visualization

**DOI:** 10.3390/s26165166

**Published:** 2026-08-14

**Authors:** Darya V. Litvinova, Ulyana S. Zubairova, Aleksandra Yu. Kravtsova

**Affiliations:** 1 Department of Physics, Novosibirsk State University, 630090 Novosibirsk, Russia; d.litvinova1@g.nsu.ru (D.V.L.); u.zubairova@g.nsu.ru (U.S.Z.); 2 Kutateladze Institute of Thermophysics SB RAS, 630090 Novosibirsk, Russia; 3 Synchrotron Radiation Facility—Siberian Circular Source of Photons “SKIF”, 630090 Novosibirsk, Russia

**Keywords:** computer vision, uncertainty analysis, cavitation, image processing, high-speed imaging, threshold correction factor, edge detection, attached-cavity length, image-based sensing, optical sensing

## Abstract

High-speed flow visualization provides imaging data containing quantitative information about cavitating-flow dynamics. Accurate determination of attached-cavity length is essential for characterizing cavitation regimes and validating mathematical models. In this study, an advanced image-processing algorithm for semi-automated analysis of cavitation patterns near hydrofoils is proposed. High-speed visualization data obtained for cavitating flow around a NACA0012 hydrofoil in a slit channel were used as input to the algorithm. The developed approach includes hydrofoil suppression, Otsu-based image binarization with threshold correction, filtering, and automated cavity-boundary detection. The initial search region for the cavity inception point is specified manually, whereas subsequent boundary tracking and cavity-length calculation are performed automatically. A dimensionless threshold correction coefficient was introduced to improve cavity identification, and its optimal range was determined. Additional geometric criteria were proposed to identify the cavity inception and closure locations and to separate attached cavities from detached vapor structures. The analysis showed that the optimal range of the threshold correction coefficient was 0.5 < th < 0.7, while a geometric connectivity criterion based on a distance of 7 px between neighboring boundary pixels provided stable detection of the cavity closure location. The developed algorithm enables the determination of both instantaneous and time-averaged attached-cavity lengths, with a total estimated uncertainty not exceeding 3.5%. Comparison with previously published experimental and analytical data demonstrated good agreement and supported the reliability of the proposed approach. The method provides an explainable and training-free computer-vision pipeline that can potentially be adapted to other bluff-body geometries under comparable imaging and contrast conditions. It can also support automated annotation and the generation of reference datasets for the development and validation of future machine-learning methods for cavitation-flow analysis.

## 1. Introduction

Cavitation occurs in flow regions where the local pressure drops below the water vapor pressure threshold [1]. The collapse of cavitation bubbles can cause erosion of hydraulic equipment surfaces and adversely affect mechanism operation [2,3]. However, cavitation shocks caused by bubble collapse can also be used to clean and modify surfaces by altering material properties [4]. For technical applications of cavitation, it is important to quickly analyze the behavior and characteristics of emerging vapor–gas cavities. A thorough investigation of cavitation characteristics can enhance its efficiency in industrial devices and minimize negative effects on hydropower equipment.

The vapor–gas cavities that occur in the flow can be either quasi-steady or unsteady [5]. A certain set of parameters is used to accurately describe the behavior of cavities and predict their further development. These include the length of the vapor–gas cavity, the time it takes for the cavity to grow and collapse, and the frequency of the cavities shedding [6,7,8]. Cavity length is treated as a key indicator of the cavitation regime, and its variations under different Reynolds, cavitation numbers and the influence of temperature are analysed [9]. Ge et al. demonstrated that variations in temperature can modify cavity length and shift the transition between attached sheet cavitation and periodic cloud shedding. This observation further supports the use of cavity extent as a quantitative variable for comparing cavitation regimes under changing operating conditions. This study focuses specifically on determining the length of an attached cavity, as this parameter is a primary indicator of the cavitation stage and directly determines the lift and drag characteristics of hydraulic profiles. Accurate characterization of cavity extent is also important for optimizing hydrodynamic cavitation reactors used in process-intensification applications. Ge et al. showed that cavitation length, cavitation intensity, and cloud-shedding behavior in a Venturi-type channel are jointly affected by the cavitation number, Reynolds number, and thermodynamic effects. Their results demonstrate the importance of robust quantitative imaging methods for evaluating cavitation performance and optimizing operating conditions [10].

The methods commonly used to determine the length of the attached cavity are high-speed visualization [11,12,13,14,15], ultrasonic pulse echography [16], pressure pulsation [17], hydrodynamic analysis based on adaptive mesh theory [18], high-speed X-ray phase contrast imaging [19,20] and particle image velocimetry (PIV) [21] with subsequent data processing. For example, advanced X-ray diagnostics can provide information inaccessible to conventional optical imaging with transparent walls. Ge et al. combined synchrotron X-ray phase-contrast imaging with edge-based tracking to obtain time-resolved velocity and cavitating fields [22]. The PIV method was used to diagnose the length of cavities by Coutier-Delgosha et al. [21]. The authors defined the area in the image without tracer particles as an attached cavity to the hydrofoil. The length of the latter represents the maximum size of the dark area in the direction of the hydrofoil chord. This method allows extracting the characteristics of the cavity at various stages of development, but with a relatively high measurement error, up to 12%.

Felici et al. [23], based on data from cavitation flow patterns obtained using both laser imaging and ultrasonic pulsed echography, employed frame-by-frame intensity averaging, followed by image processing and approximation of the bubble boundary with polynomial functions. An area with an intensity above 80% of the maximum brightness of the image was taken as the boundary of the cavity. Hutli et al. [24] detected cavitation clouds by isolating their boundaries in a binary image after applying a smoothing algorithm. They then contrasted the cavitation clouds in the image using ellipses and performed a time analysis on the resulting metrics. However, these image analysis techniques have a high measurement error that can reach 15%. Therefore, it is important to develop alternative methods for accurately determining the length of vapor–gas cavities.

High-speed visualization can be considered a particular type of imaging sensing, where image sequences contain quantitative information about rapidly evolving physical processes. In such applications, computer-vision techniques provide efficient tools for extracting geometric and temporal characteristics from experimental data while preserving physical interpretability. Visualization of cavitation flows and frame-by-frame video processing are among the most promising research methods. There are various techniques used to enhance the efficiency of image processing of high-speed flow visualization data, such as increasing the number of gray levels and blurring or smoothing the image with a median filter [25,26,27,28]. The analysis methods presented in the literature so far determine only the approximate position of the vapor–gas bubbles and cavities in the image. At the same time, when boundaries are detected using color gradients between neighboring pixels, the boundaries of the attached cavity and the cavitation cloud present in the flow may merge. This can complicate or completely preclude the quantification of the dimensional characteristics of the attached cavities. Determining additional criterial parameters and image processing components would increase the accuracy of searching for vapor–gas cavities and bubbles in an image, reducing the time required to analyze images of cavitation flows, as well as the time for detecting both the average length of the attached cavity and the length of the attached cavity at each of the studied time points.

Recent advances in deep learning have shown remarkable success in image segmentation and object detection tasks [29,30], with applications extending to fluid flow analysis [31,32,33]. For example, Liu et al. used a U-Net-based model to identify cavitation regions and calculate their geometric characteristics [34]. However, the construction of such models requires pixel-level annotated masks, and their performance depends on how well the training data represent variations in illumination, hydrofoil geometry, spatial resolution, and cavitation regime. Generating sufficiently diverse annotations from high-speed experiments is labor-intensive [35]. Consequently, a training-free and physically interpretable pipeline remains valuable both as an independent measurement method and as a tool for generating reference annotations for subsequent machine-learning studies. Classical image-processing approaches remain valuable for several reasons: (1) they provide interpretable results, allowing each processing step to be evaluated in relation to the underlying physical process; (2) they require no training data and can therefore be applied directly to new experimental configurations; and (3) they provide computationally efficient offline batch processing of high-speed flow-visualization data. Moreover, accurate classical image-processing methods can provide explainable reference solutions and facilitate the automated annotation of large experimental datasets, thereby supporting the development and validation of future machine-learning models for cavitation analysis.

The goal of this study is to develop an explainable computer-vision framework for extracting quantitative characteristics of attached cavities from high-speed visualization data and to identify robust criteria that improve measurement accuracy. The experimental acquisition of high-speed cavitation-flow images is first described, followed by a detailed presentation of the automated image-processing algorithm. Particular attention is given to the selection and validation of the coefficients and threshold values used at different processing stages. The performance of the proposed approach is then demonstrated by measuring attached-cavity length for cavitating flow around a hydrofoil in a slit channel. Previous optical image-processing approaches have reported measurement errors of approximately 12–15% and may fail when the boundary of an attached cavity merges with detached vapor clouds. The proposed method addresses two related failure modes: fragmentation of the cavity region during thresholding and erroneous continuation of the tracked boundary into detached vapor structures. These issues are mitigated using an Otsu-derived threshold correction coefficient and a calibrated geometric connectivity criterion. The resulting total estimated uncertainty in the attached-cavity-length measurements did not exceed 3.5%, which is substantially lower than the error ranges reported for previous approaches. In addition, the results of the semi-automated analysis were manually verified frame by frame for approximately 15,000 frames representing the cavitation regimes considered.

## 2. Materials and Methods

### 2.1. Experimental Setup

Data on the high-speed visualization of cavitating flows were obtained in a slit channel of a closed hydrodynamic circuit. The working section consisted of two parallel transparent plates forming a slit channel with a length of 245 mm, a height of 120 mm, and a depth of 1.4 mm. A body with a NACA 0012 hydrofoil profile (National Advisory Committee for Aeronautics, NACA) was installed in the central region of the channel. The span of the hydrofoil was 1.4 mm and was equal to the depth of the slit channel. The angle of attack of the hydrofoil, α, was 8°; the chord length, *C*, was 70 mm; and the average surface roughness of the hydrofoil was 30 µm.

The experiments were carried out using distilled water. To reduce the amount of dissolved gas, the water was degassed before the measurements by increasing the flow rate and subsequently removing the released air bubbles from the upper reservoir using a vacuum pump. The amount of dissolved air in the experiments did not exceed 0.023 g/kg. The flow rate was measured using a “VZLET” flowmeter (JSC “VZLET”, Saint Petersburg, Russia) integrated into the experimental loop. The measurement error did not exceed 2%. The bulk flow velocity in the working section was calculated from the measured flow rate and the effective cross-sectional area of the slit channel. The bulk velocity, $U_{\infty }$, varied from 5 to 22 m/s. A cooling circuit was used to maintain a constant liquid temperature during the experiments. The water temperature was 27 °C. The pressure at the inlet of the working section was measured using an SDV-IV sensor (JSC “NPK VIP”, Yekaterinburg, Russia) with an accuracy of ±0.5%. The inlet pressure reached 350 kPa. A more detailed description of the experimental setup can be found in Ref. [5].

The Reynolds number was defined as

(1) $$Re=\frac{U_{\infty }C}{\nu },$$

where $U_{\infty }$ is the bulk flow velocity, *C* is the hydrofoil chord length, and ν is the kinematic viscosity of water.

The cavitation number was defined as

(2) $$\sigma =\frac{p_{\infty }-p_{v}}{0.5\rho U_{\infty }^{2}},$$

where $p_{\infty }$ is the characteristic pressure of the incoming flow, $p_{v}$ is the saturated vapor pressure, and ρ is the liquid density.

During the experimental studies, the cavitation number varied from 1.98 to 1.30, which allowed us to investigate cavitating flows over a wide range of operating conditions. Lower values of the cavitation number correspond to a higher probability that the local pressure decreases to the saturated vapor pressure and, consequently, to a higher probability of cavitation inception and development.

**Figure 1:**
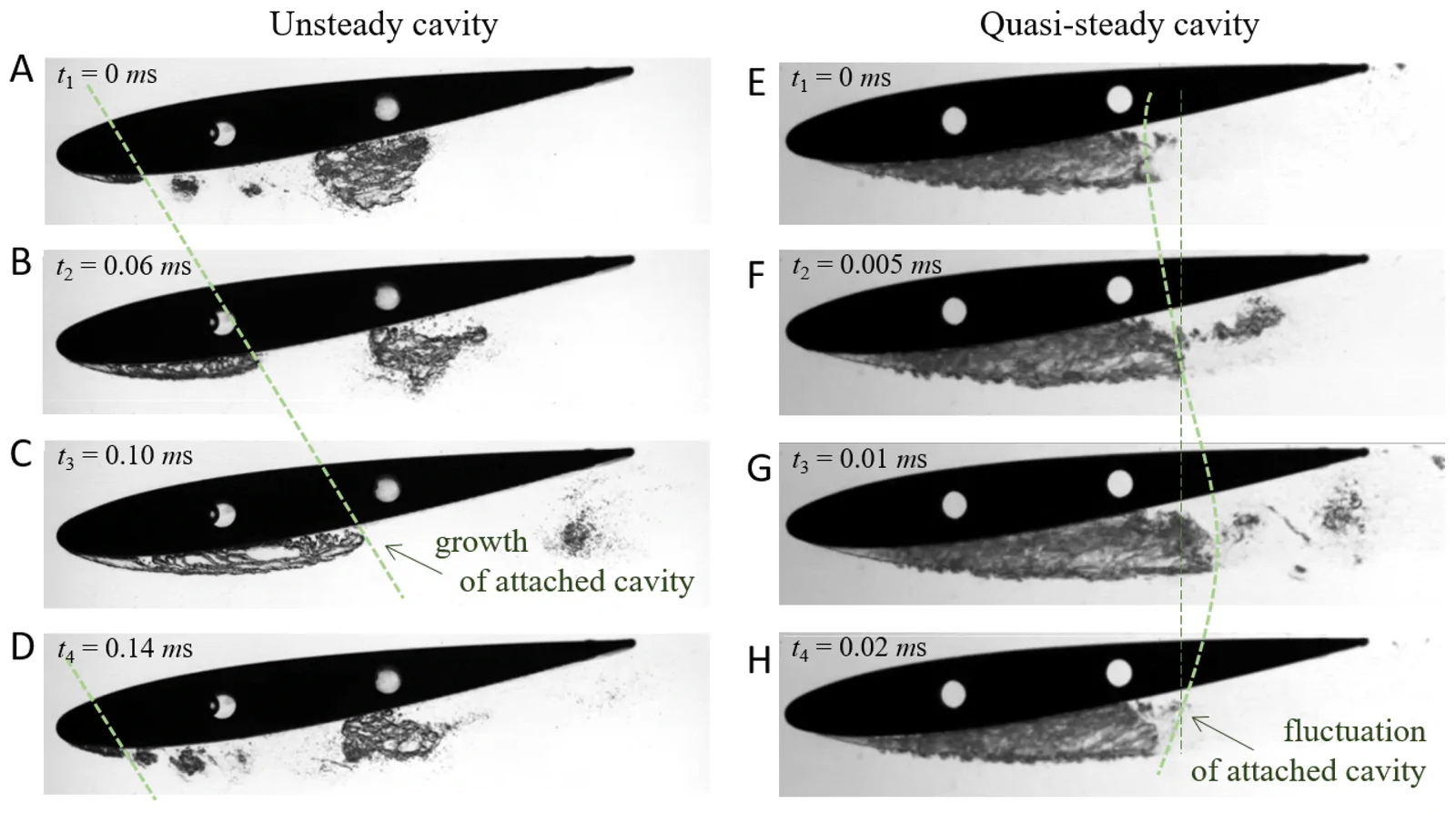
High-speed visualization of the cavitating hydrofoil for (**A**–**D**) unsteady regime, $Re=1.1\times 10^{6}$, σ=1.55, and (**E**–**H**) quasi-steady regime, $Re=1.42\times 10^{6}$, σ=1.44.

A high-speed visualization method was used to capture the cavitating flow. Images were recorded using a Photron Fastcam Nova S12 camera (Photron Ltd., Tokyo, Japan) with a frame rate of 20 kHz. Uniform back illumination was provided by an LED light source equipped with a diffuser. The luminous flux of the lamp was 20,000 lumens. A time sequence of the high-speed visualization data is shown in Figure 1.

### 2.2. Image Processing Algorithm

To determine the length of the attached cavity, a multi-stage image-processing algorithm was developed and implemented in the MATLAB environment. The first stage of processing is the removal of the hydrofoil image from the frame. For unsteady cavitation regimes, the background image can be obtained as a temporal average over the entire video sequence and subsequently subtracted from each frame. This approach does not require a separate image of the undisturbed background and can be applied directly to ordinary high-speed recordings. The averaged image is calculated as

(3) $$I_{\mathrm{aver}}=\frac{1}{N}\sum_{k=1}^{N}I_{k},$$

where $I_{k}$ is the *k*-th frame and *N* is the total number of frames. The image after background subtraction is determined as

(4) $$I_{\mathrm{rez},k}=I_{k}-I_{\mathrm{aver}}.$$

However, in the case of quasi-steady cavitation, simple subtraction of the averaged image may remove a portion of the attached cavity itself and complicate the determination of its instantaneous length. A separate reference image of the hydrofoil without flow could simplify this procedure, but such images are not always available when previously acquired experimental data are analyzed. In addition, small vibrations of the hydrofoil image were observed at high Reynolds numbers, which could leave residual hydrofoil pixels after direct subtraction of a single reference image. Therefore, an additional procedure for hydrofoil detection and suppression was introduced.

The hydrofoil was identified by selecting pixels with intensities between 85% and 100% of the maximum image intensity. Relative thresholds close to the maximum intensity have previously been used in image-analysis applications to retain high-confidence or representative pixels of a target object or image feature [36,37,38]. In cavitation visualization, Felici et al. similarly used a threshold corresponding to approximately 80% of the maximum image brightness during cavity-boundary reconstruction [23]. Such threshold values are application-specific rather than universal; in the present study, the lower bound of 85% was selected empirically under the applied illumination conditions and provided stable hydrofoil detection in the presence of small image displacements. The hydrofoil contour, $I_{c}$, was additionally detected using the Sobel operator implemented through the edge function. The intensity of the object boundary differed slightly from that of the hydrofoil body, and the thickness of this boundary region was approximately 1–4 px. Combining relative intensity thresholding with edge detection therefore enabled more complete suppression of the hydrofoil image.

Small residual pixel clusters originating from incomplete boundary removal and local image non-uniformities were eliminated using the bwareafilt function. The corrected image and the original averaged image were subsequently smoothed using a two-dimensional median filter. This procedure reduced the influence of the hydrofoil body, shadow regions, and isolated artifacts.

**Figure 2:**
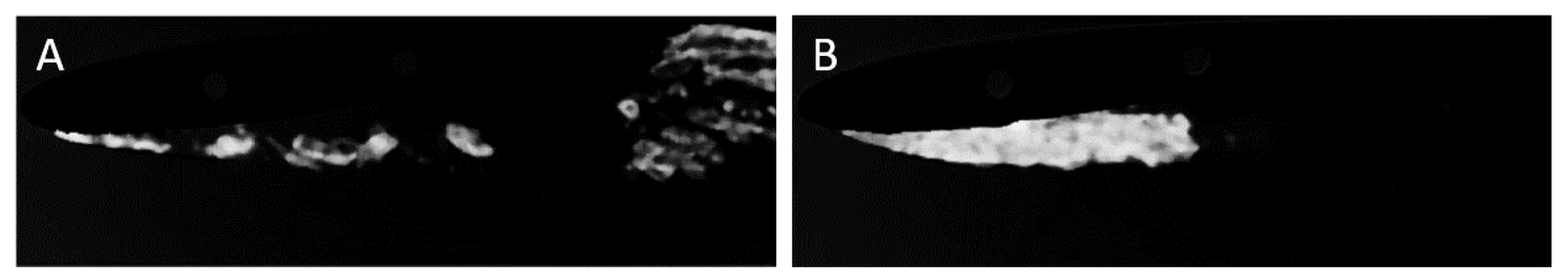
Examples of images after hydrofoil suppression for (**A**) unsteady and (**B**) quasi-steady cavitation regimes.

Examples of the processed images obtained after hydrofoil suppression are shown in Figure 2. The proposed preprocessing procedure makes the algorithm applicable to cavitation visualization data obtained near bluff bodies of arbitrary shape. The detailed implementation of the hydrofoil suppression procedure is presented in Appendix A.

The RGB images were converted into grayscale using the im2gray function. Image binarization was performed using Otsu’s method [39]. First, the threshold value was determined independently for each frame and then averaged over the entire video sequence. Otsu’s method determines the threshold from the image intensity histogram by maximizing the separation between foreground and background classes.

To improve cavity detection accuracy, a dimensionless threshold correction coefficient, denoted as th, was introduced. The threshold used for image binarization was defined as

(5) $$T=th\cdot T_{\mathrm{Otsu}},$$

where $T_{\mathrm{Otsu}}$ is the averaged threshold obtained using Otsu’s method. The threshold correction coefficient was varied from 0.2 to 0.9. For each value of th, the longitudinal size of the continuous white region, λ, was measured and compared with the original visualization data. High threshold values produced artificial ruptures in the cavity boundary, whereas excessively low values caused neighboring structures to merge.

The analysis showed that the interval 0.5<th<0.7 provides nearly invariant cavity dimensions and minimizes the measurement error. Within this range, the uncertainty associated with image binarization did not exceed 3.5%.

To suppress isolated pixels and detached vapor structures, the binary image was additionally processed using the two-dimensional median filter medfilt2. The outer cavity boundary was then determined automatically. Starting from the cavity nucleation point, the algorithm searched for boundary pixels with the maximum coordinate along the $O_{y}$ axis for each position along the $O_{x}$ axis.

The nucleation point was selected manually only once at the beginning of the analysis and was subsequently used as the starting point for automated boundary tracking. The boundary search was terminated when the distance between neighboring white pixels exceeded 7 px along either coordinate axis. Under the present experimental conditions, this threshold corresponded to approximately 0.54 mm and was used to distinguish detached cavitation clouds from the attached cavity. The first detected boundary point was considered the cavity nucleation point, whereas the last point corresponded to the cavity closure location.

The cavity length was defined as the shortest distance between these two points. For each video sequence, the hydrofoil chord length in pixels was measured and compared with the known physical chord length. The corresponding scale factor was used to convert cavity dimensions from pixels to millimeters. The average cavity length was calculated as

(6) $$l_{\mathrm{avg}}=\frac{1}{N}\sum_{i=1}^{N}l_{i},$$

where $l_{i}$ is the instantaneous cavity length in the *i*-th frame.

The uncertainty analysis considered the contributions of image binarization and spatial calibration. The initial search region for the cavity leading edge was specified manually, whereas the actual inception and closure points were subsequently determined automatically from the binarized image. Because these points were obtained as the extreme coordinates of the segmented cavity region, the numerical search itself introduced no additional uncertainty relative to the binary mask; uncertainty in their positions was therefore included in the binarization error. The hydrofoil-suppression mask was used only during image preprocessing and did not directly enter the cavity-length calculation; its contribution was considered negligible, provided that the cavity region was preserved after masking. The dominant source of uncertainty was image binarization, which contributed approximately 3.5%. The spatial calibration was based on a hydrofoil chord spanning approximately 800 pixels; an uncertainty of 2–3 pixels in the chord measurement corresponded to approximately 0.3%. Assuming that the binarization and calibration contributions were independent, the combined uncertainty was approximately 3.5%.

Using MATLAB R2014b on a personal computer equipped with an Intel Core i7-11800H processor (2.30 GHz), the proposed algorithm processed a sequence of 5000 frames in 28.37 s, corresponding to an average processing time of 5.67 ms per frame and a throughput of approximately 176 frames s^−1^.

## 3. Results

### 3.1. Cavitation Regimes Observed in High-Speed Visualization

A high-speed visualization technique made it possible to investigate the evolution of attached cavities on the suction side of the NACA0012 hydrofoil under different flow conditions. Depending on the Reynolds number and cavitation number, two characteristic cavitation regimes were observed: an unsteady cavity and a quasi-steady cavity. Representative time sequences corresponding to these regimes are shown in Figure 1.

The gas–vapor cavity on the suction side of the hydrofoil in the slit channel can take two characteristic states: an unsteady cavity (Figure 1A–D) and a quasi-steady cavity (Figure 1E–H). The length of the unsteady cavity varies significantly over time, with gradual growth followed by rapid collapse of the attached cavity until its disappearance from the flow. After that, the process repeats. A characteristic feature of the quasi-steady cavity is its continuous presence on the hydrofoil surface. In this regime, only slight variations in cavity length are observed in the closure region. These two flow regimes were subsequently used to assess the applicability of the proposed image-processing algorithm under substantially different cavitation conditions.

### 3.2. Hydrofoil Suppression and Image Preprocessing

The first stage of the proposed algorithm consists in removing the hydrofoil image from each frame of the high-speed visualization sequence. This preprocessing step allows subsequent cavity detection to be performed independently of the hydrofoil geometry and reduces the influence of stationary image components. Examples of images obtained after applying the hydrofoil suppression procedure are presented in Figure 2.

As can be seen from Figure 2, the preprocessing procedure removes the hydrofoil image while preserving the shape and boundaries of the vapor–gas cavity. The resulting images contain only cavitation structures, which simplifies further binarization and boundary detection.

Preprocessing in the form of hydrofoil suppression makes the proposed algorithm applicable to high-speed visualization data obtained in the vicinity of bluff bodies of arbitrary geometry. The procedure therefore increases the universality of the algorithm and allows it to be used for both unsteady and quasi-steady cavitation regimes.

### 3.3. Effect of Threshold Correction Coefficient

The accuracy of cavity detection strongly depends on the threshold used for image binarization. The average threshold value obtained using Otsu’s method is often too high to completely binarize the pixels corresponding to the vapor–gas cavity. As a result, artificial ruptures may appear along the outer cavity boundary, leading to an increased error in determining the cavity length.

To improve the quality of cavity detection, the threshold correction coefficient, th, was introduced. The coefficient was varied within the range

th=0.2,0.3,0.4,0.5,0.55,0.6,0.65,0.7,0.8,0.9.

**Figure 3:**
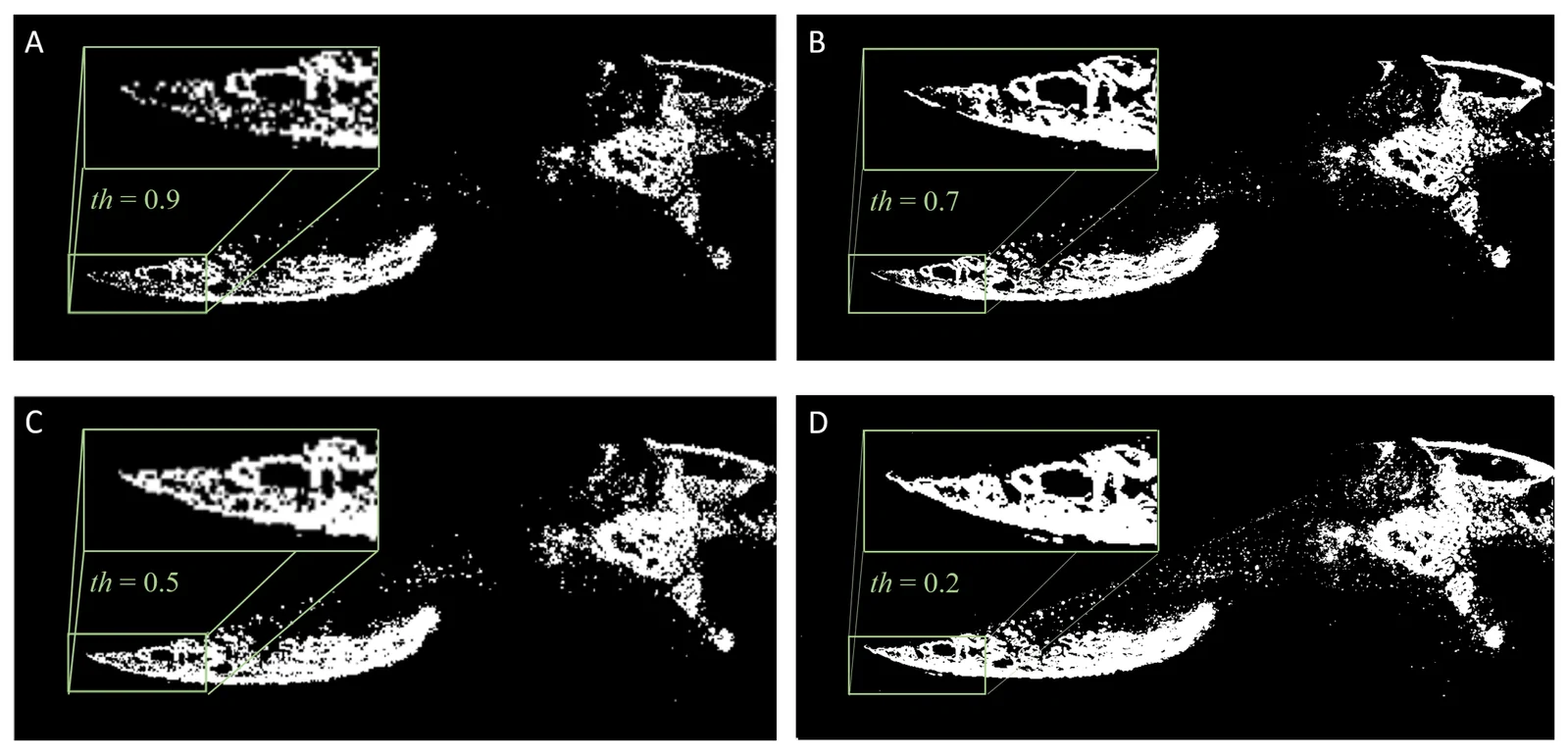
Examples of binarized images obtained using different threshold correction coefficients: (**A**) th=0.9, (**B**) th=0.7, (**C**) th=0.5, and (**D**) th=0.2.

Examples of binarized images obtained for different values of the threshold correction coefficient are presented in Figure 3.

**Figure 4:**
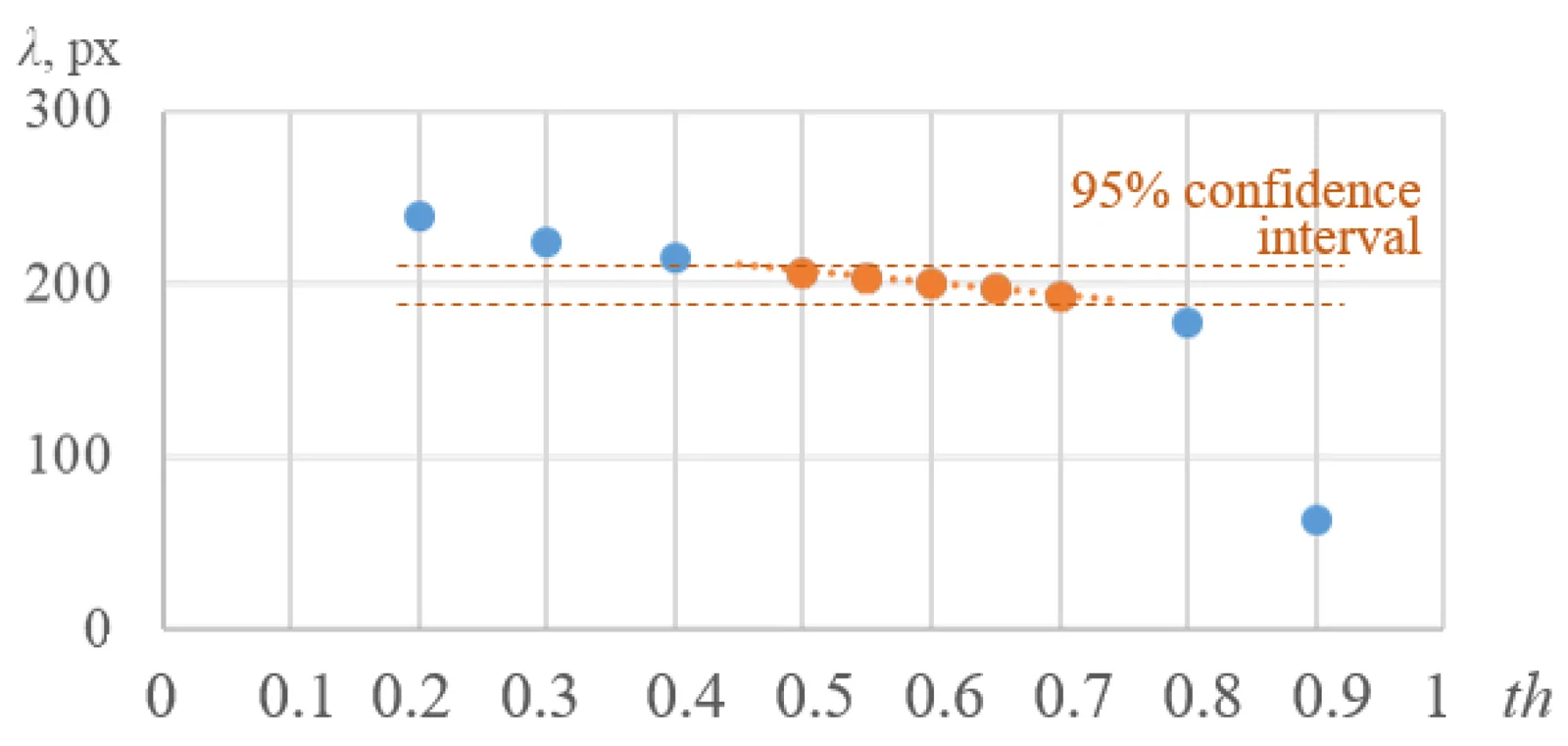
Dependence of the longitudinal size of the continuous white region, λ, on the threshold correction coefficient, th. Orange dots indicate measurements within the selected stable interval (0.5<th<0.7), whereas blue dots indicate measurements outside this interval. The flow parameters are $Re=1.1\times 10^{6}$ and σ=1.55. λ=201 px indicates the median absolute length error normalized by the hydrofoil chord, while the two outer dashed lines indicate the corresponding 95% bootstrap confidence interval. The interval 0.5<th<0.7 corresponds to the region of stable cavity-length measurements.

At high values of the threshold correction coefficient (Figure 3A), the distances between neighboring white pixels may exceed 7 px and are therefore incorrectly interpreted by the algorithm as regions without a vapor–gas cavity. Consequently, the detected cavity length becomes underestimated. In contrast, excessively low values of the threshold correction coefficient cause neighboring structures to merge, resulting in an artificial increase in the detected cavity dimensions (Figure 3D). For each value of th, the longitudinal size of the white region with a continuous boundary, λ, was measured and compared with the original high-speed visualization data. The dependence of λ on the threshold correction coefficient is shown in Figure 4.

A region of nearly invariant measurements is observed for 0.5<th<0.7. These values were obtained using confidence intervals statistical methods. The median absolute length error normalized by the hydrofoil chord, together with 95% bootstrap confidence intervals. Within this interval, the size of the selected region remains almost constant and equals approximately 201±7 px at $Re=1.1\times 10^{6}$. At higher values of the threshold correction coefficient (th>0.7), the longitudinal size of the detected cavity decreases sharply. For example, at th=0.9, the measured value λ=64 px, which is approximately three times smaller than the values obtained in the self-similar region.

Therefore, the optimal range of threshold correction coefficients is 0.5<th<0.7. Within this range, the uncertainty associated with image binarization does not exceed 3.5%.

### 3.4. Boundary Detection Criteria

**Figure 5:**
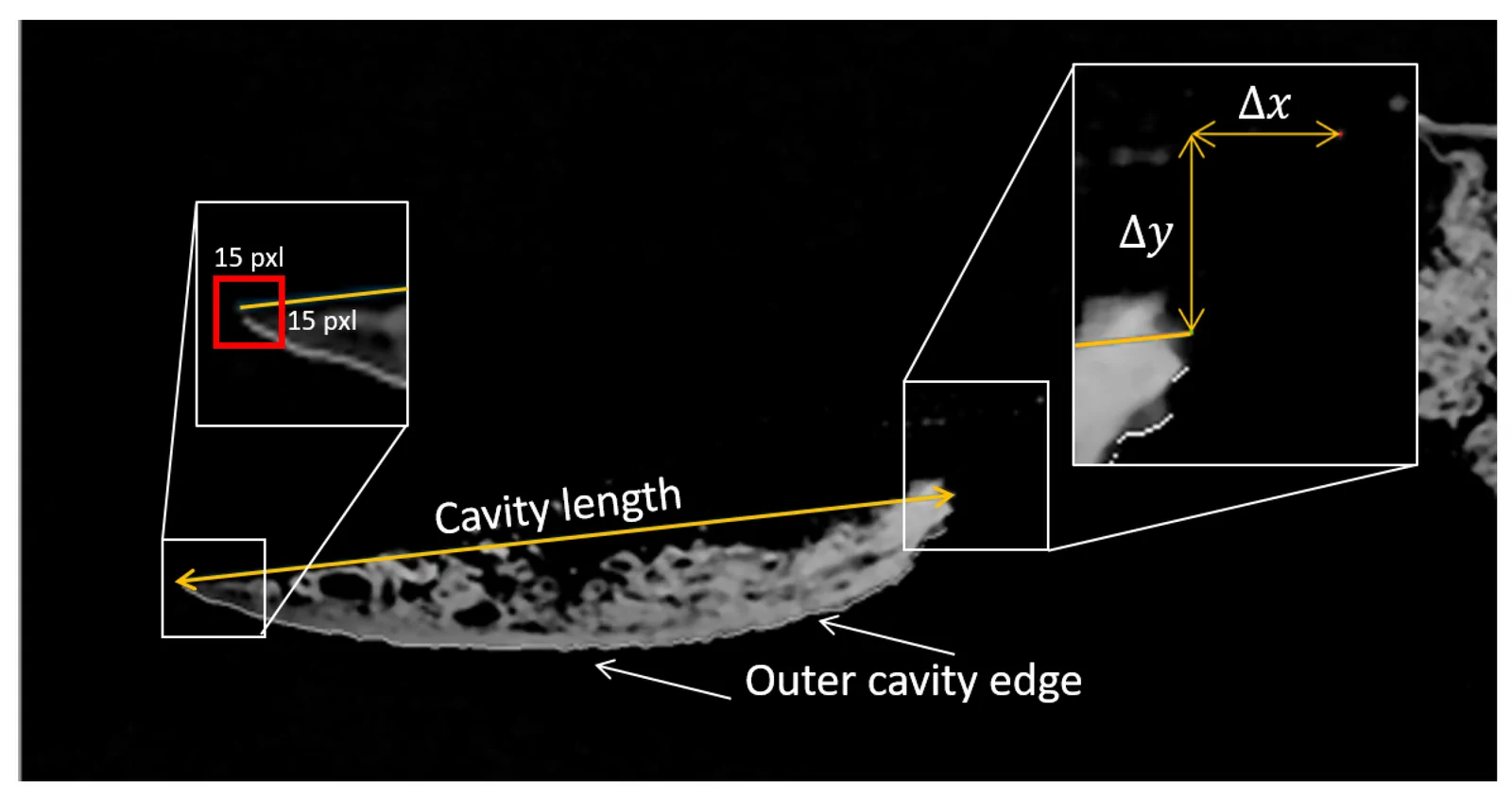
Criteria used to determine the leading edge and closure location of the attached cavity. The red box indicates a spatial scale of 15 px.

Accurate determination of the cavity length requires reliable identification of both the nucleation point and the closure location of the attached cavity. For this purpose, additional geometric criteria were introduced into the image-processing algorithm. The criterion used to determine the leading edge and closure location of the attached cavity is illustrated in Figure 5.

Starting from the cavity nucleation point, the algorithm sequentially searches for the white pixel having the maximum coordinate along the $O_{y}$ axis for each subsequent position along the $O_{x}$ axis. The search is performed using the standard find function, which returns the coordinates of the boundary pixels.

Before the search begins, an empty array is initialized to store the coordinates of the detected boundary points. The array is then filled sequentially with the coordinates of pixels belonging to the outer cavity boundary. The boundary search is terminated when the distance between two neighboring white pixels exceeds 7 px along either the $O_{x}$ or $O_{y}$ axis. Under the present experimental conditions, this threshold corresponds to approximately 0.54 mm and allows detached cavitation clouds to be distinguished from the attached cavity. The first element of the coordinate array is considered the cavity nucleation point, whereas the last element corresponds to the cavity closure location. The cavity length is then defined as the shortest distance between these two points. The introduced criteria make it possible to separate the attached cavity from detached vapor structures and improve the robustness of cavity length measurements for both unsteady and quasi-steady cavitation regimes.

### 3.5. Instantaneous Cavity Length

**Figure 6:**
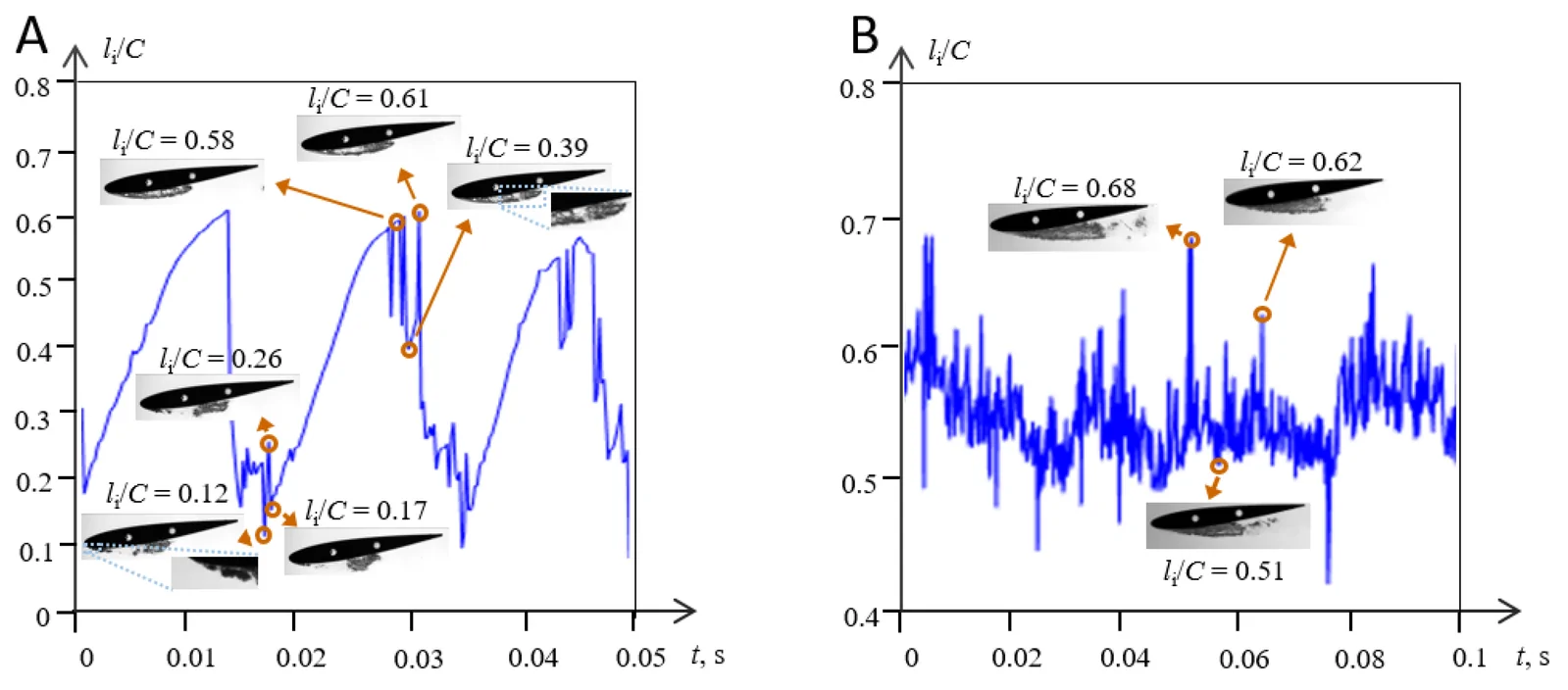
Temporal evolution of the normalized attached cavity length for (**A**) an unsteady cavitation regime, $Re=1.1\times 10^{6}$, σ=1.55, and (**B**) a quasi-steady cavitation regime, $Re=1.42\times 10^{6}$, σ=1.44. Corresponding flow-visualization images are shown for selected characteristic time points at which the cavity length was manually verified.

Application of the proposed image-processing algorithm to high-speed visualization data made it possible to determine the temporal evolution of the attached cavity length for different flow conditions. Examples of the normalized cavity length as a function of time for unsteady and quasi-steady cavitation regimes are presented in Figure 6.

Figure 6A shows the behavior of an unsteady cavity. In this regime, the cavity growth time considerably exceeds the collapse time. The cavity develops gradually until reaching its maximum length, after which rapid collapse of the vapor–gas cavity occurs. In contrast, the quasi-steady regime shown in Figure 6B is characterized by the continuous presence of the attached cavity on the hydrofoil surface. The cavity length remains relatively stable over time, although short-time fluctuations of the normalized cavity length can be observed. These sudden variations are associated with the physical dynamics of the closure region. Detached cavitation clouds may separate from the attached cavity and subsequently merge with it again. Therefore, the observed peaks reflect the actual dynamics of the cavitating flow rather than random measurement noise. The developed algorithm makes it possible to determine the instantaneous cavity length for both unsteady and quasi-steady cavitation regimes with a total measurement uncertainty not exceeding 3.5%.

## 4. Discussion

The average cavity length is one of the most commonly used parameters for describing cavitating flows, comparing experimental results, and validating mathematical models. The average cavity length was calculated as

(7) $$l_{\mathrm{avg}}=\frac{1}{N}\sum_{i=1}^{N}l_{i},$$

where $l_{i}$ is the attached cavity length measured in the *i*-th frame and *N* is the total number of frames. The results of calculating the average cavity length for different cavitation regimes are summarized in Table 1.

The average cavity length is often used to characterize cavitating flows, compare research results, and validate mathematical models. Brennen [40] obtained an analytical relationship for a two-dimensional infinitely thin plate:

(8) $$\frac{\sigma }{2\alpha }=\frac{2-L/C+2\sqrt{1-L/C}}{\left(L/C\right)\sqrt{1-L/C}},$$

where σ is the cavitation number, α is the angle of attack expressed in radians, *L* is the attached cavity length, and *C* is the hydrofoil chord length.

Another approach for comparing attached cavity lengths obtained for different hydrofoils and experimental facilities was proposed by Franc [41]. According to this scaling law,

(9) $$\frac{L}{C}∼{\left(\frac{\sigma }{2\alpha }\right)}^{-1}.$$

**Figure 7:**
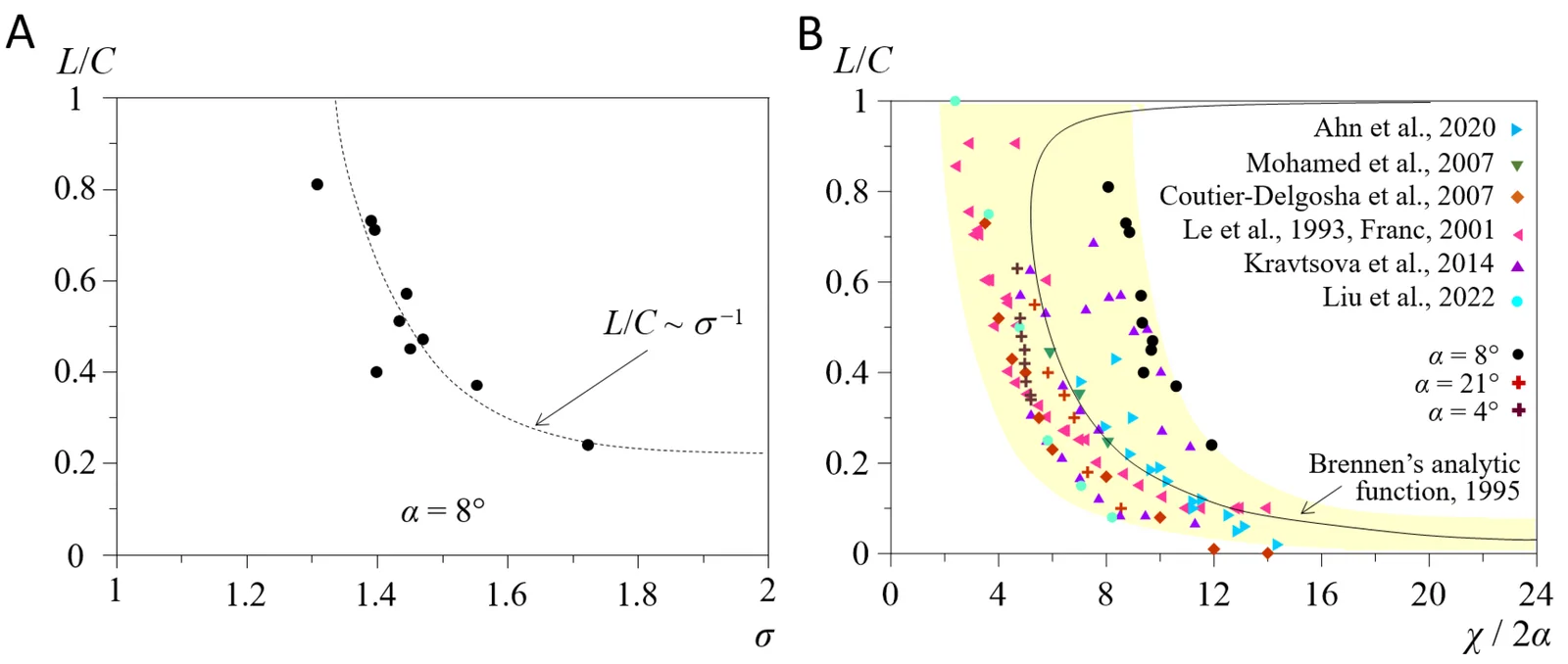
(**A**) Dependence of the normalized cavity length on the cavitation number obtained using the proposed image-processing algorithm. (**B**) Comparison of the present results with literature data and analytical predictions from [40], Ahn et al. [42], Mohamed et al. [43], Coutier-Delgosha et al. [21], Le et al. [44], Franc [41], Kravtsova et al. [46], and Liu et al. [45].

The dependence of the normalized cavity length on the cavitation number obtained in the present study is shown in Figure 7A.

Figure 7B compares the present measurements with the results reported previously for various hydrofoil geometries and flow conditions [6,11,21,41,42,43,44,45,46]. The literature data include NACA0012, NACA0015, plano-convex, and S-shaped hydrofoils investigated at different angles of attack. Our research data are represented in Figure 7B by black circles for the angle of attack of 8°, by burgundy crosses for 21° [6], and by brown crosses for 4°.

To compare the results obtained in the slit channel with data from conventional hydrodynamic facilities, the criterion introduced by Kravtsova et al. [6] was used. For large-scale hydrodynamic facilities, the parameter χ is equal to the cavitation number,

(10) χ=σ.

For slit channels,

(11) $$\chi =\sigma \epsilon \frac{l}{D},$$

where ϵ is the friction coefficient determined using the Blasius equation,

(12) $$\epsilon =\frac{0.347}{Re_{D}^{0.25}}.$$

The black circles and crosses corresponding to the present measurements lie within the yellow region proposed in Ref. [6]. The obtained results demonstrate good agreement with previously published experimental and analytical data and confirm the high quality and reliability of the proposed image-processing algorithm for cavitating-flow analysis.

## 5. Conclusions

The paper presents an advanced image-processing algorithm for automatic analysis of cavitation patterns near hydrofoils. The method was applied to cavitating flows generated in a slit tunnel with a NACA0012 hydrofoil positioned at an angle of attack of 8°. Depending on the Reynolds number and cavitation number, unsteady and quasi-steady cavities were observed on the suction side of the hydrofoil.

High-speed visualization data obtained in the experiments were used as the initial input for the image-processing procedure. A preprocessing stage for hydrofoil suppression was developed, followed by image binarization, filtering, and automated boundary detection. The optimal range of the threshold correction coefficient was determined, and additional criteria for identifying the cavity closure location were introduced.

The proposed algorithm separates attached cavities from detached vapor structures by applying a geometric criterion based on the distance between neighboring boundary pixels. Under the present experimental conditions, a threshold value of 7 px (approximately 0.54 mm) provided stable determination of the cavity closure location.

The developed approach made it possible to determine both instantaneous and time-averaged cavity lengths with a total uncertainty not exceeding 3.5%. Comparison with previously published experimental and analytical data demonstrated good agreement and confirmed the reliability of the proposed image-processing algorithm.

The proposed approach can be applied to cavitation flows around bluff bodies of arbitrary geometry and represents an explainable and training-free computer-vision framework for extracting quantitative information from dynamic imaging data acquired by high-speed vision sensors. In addition to direct measurements, the algorithm can be used for automated annotation and generation of reference datasets required for the development, training, and validation of future machine-learning models for cavitation-flow analysis.

## Figures and Tables

**Table 1:** Changes in the length of the attached cavity with increasing flow rate. Re is the Reynolds number; *Q* is the volumetric flow rate; σ is the cavitation number; *L* is the average length of the attached vapor–gas cavity; L/C is the normalized dimensionless average cavity length; *C* is the chord length of the NACA0012 hydrofoil.

Re·106	*Q*, m^3^/h	σ	*L*, px	*L*, mm	L/C
1.04	7.1	1.72	218	16.8	0.24
1.10	7.5	1.55	337	25.9	0.37
1.21	8.3	1.45	409	31.5	0.45
1.26	8.6	1.47	428	32.9	0.47
1.34	9.2	1.43	464	35.7	0.51
1.42	9.7	1.44	519	39.9	0.57
1.51	10.3	1.40	646	49.7	0.71
1.58	10.8	1.39	664	51.1	0.73
1.70	11.6	1.30	737	56.7	0.81

## Data Availability

The data that support the findings of this study are available from the corresponding author upon reasonable request.

## Abbreviations

The following abbreviations are used in this manuscript:

σ	cavitation number
Re	Reynolds number
α	angle of attack of the hydrofoil or bluff body
*L*	length of the attached vapor–gas cavity
*C*	chord length of the hydrofoil or bluff body
$p_{\infty }$	characteristic pressure of the incoming flow
$p_{v}$	saturated vapor pressure
ρ	liquid density
$U_{\infty }$	bulk/free-stream flow velocity
*Q*	volumetric flow rate
$I_{k}$	*k*-th instantaneous image/frame in the video sequence
*N*	number of frames in the video sequence
$I_{\mathrm{aver}}$	temporal-average image used as the background image
$I_{\mathrm{th}}$	thresholded image containing pixels above the selected intensity threshold
$I_{c}$	hydrofoil-contour image obtained by edge detection
$I_{\mathrm{bwl}}$	binary mask of residual pixel clusters selected by area filtering along the hydrofoil contour
$I_{\mathrm{rez},k}$	*k*-th processed image after subtraction/removal of the bluff-body image
th	dimensionless threshold correction coefficient used for image binarization
λ	longitudinal size of the continuous white region in a binarized image, px
ϵ	the friction coefficient determined using the Blasius equation
$l_{i}$	instantaneous attached-cavity length in the *i*-th frame
$l_{\mathrm{avg}}$	time-averaged attached-cavity length over a video sequence
px	pixel unit used for image-coordinate measurements
$O_{x}$, $O_{y}$	image-coordinate axes used for boundary search

## Appendix A. Preprocessing Procedure for Hydrofoil Suppression

The algorithm used in this study to determine the length of the attached vapor–gas cavity is presented below.

**Algorithm A1** Preprocessing procedure for hydrofoil-region suppression
**Input:** Averaged grayscale image *I* **Output:** Residual image *D* 1. Store the original image: $$I_{0}\leftarrow I$$ 2. Determine the maximum image intensity: $$I_{\max}\leftarrow \max\left(I_{0}\right)$$ 3. Extract high-intensity pixels: $$I_{\mathrm{th}}\left(x,y\right)\leftarrow I_{0}\left(x,y\right),\;I_{0}\left(x,y\right)\geq 0.85I_{\max}$$ otherwise $$I_{\mathrm{th}}\left(x,y\right)\leftarrow 0$$ 4. Detect image edges using the Sobel operator: $$E\leftarrow \mathrm{Sobel}(I_{0})$$ 5. Suppress the hydrofoil region and its edges: $$I_{w}\leftarrow I_{0}-I_{\mathrm{th}}-E$$ 6. Binarize the resulting image: $$B\leftarrow \mathrm{Binarize}(I_{w})$$ 7. Retain the two largest connected components: $$B_{2}\leftarrow \mathrm{AreaFilter}\left(B,2\right)$$ 8. Identify the removed small components: $$R\leftarrow B-B_{2}$$ 9. Set pixels corresponding to *R* to zero in $I_{w}$. 10. Apply median filtering to $I_{w}$ using a 6×6 window. 11. Apply median filtering to $I_{0}$ using a 6×6 window. 12. Compute the residual image: $$D\leftarrow \mathrm{MedianFilter}\left(I_{0}\right)-\mathrm{MedianFilter}\left(I_{w}\right)$$ 13. Return *D*.
